# AI‐ECG for early detection of atrial fibrillation: First‐year results from a stroke prevention study in Shimizu, Japan

**DOI:** 10.1002/joa3.70132

**Published:** 2025-07-04

**Authors:** Mayumi Masumura, Atsuyuki Ohno, Haruhiko Yoshinaga, Takeshi Sasaki, Yasuteru Yamauchi, Hitoshi Hachiya, Atsushi Takahashi, Yasushi Imai, Hideo Fujita, Kensuke Ihara, Yusuke Ebana, Toshihiro Tanaka, Tetsushi Furukawa, Tetsuo Sasano

**Affiliations:** ^1^ Department of Cardiology Shizuoka City Shimizu Hospital Shizuoka Japan; ^2^ Shizuoka City Shimizu Medical Association Shizuoka Japan; ^3^ Department of Cardiology NHO Disaster Medical Center Tokyo Japan; ^4^ Department of Cardiology Japan Red Cross Yokohama City Bay Hospital Yokohama Kanagawa Japan; ^5^ Cardiovascular Center Tsuchiura Kyodo Hospital Ibaraki Japan; ^6^ Department of Cardiology Yokosuka Kyosai Hospital Yokosuka Kanagawa Japan; ^7^ Division of Clinical Pharmacology and Division of Cardiovascular Medicine Jichi Medical University Shimotsuke Japan; ^8^ Department of Cardiovascular Medicine Jichi Medical University Saitama Medical Center Saitama Japan; ^9^ Department of Cardiovascular Medicine Institute of Science Tokyo Tokyo Japan; ^10^ Department of Medical Genetics Institute of Science Tokyo Tokyo Japan; ^11^ Department of Human Genetics and Disease Diversity Institute of Science Tokyo Tokyo Japan; ^12^ Institute of Science Tokyo Tokyo Japan

**Keywords:** artificial intelligence, atrial fibrillation, cohort study, electrocardiogram, screening

## Abstract

**Background:**

An artificial intelligence algorithm‐guided electrocardiogram (AI‐ECG) has been developed to detect atrial fibrillation (AF) in patients with sinus rhythm (SR). However, its utility for population‐based screening remains unclear in Japan.

**Method and Results:**

In this prospective cohort study, “SPAFS” (Stroke Prevention by Early Detection of AF in Shimizu), participants who underwent health examinations at the Shimizu Medical Association Examination Center from January 2022 to July 2023 were enrolled, with known AF excluded. ECGs were categorized by AI as low‐, moderate‐, or high risk: non‐SR were labeled as non‐applicable (NA). All participants underwent 7‐day single‐lead ECG monitoring. Among 362 participants (61.1 ± 10.5 years, 38% male, CHADS2 score 0.49 ± 0.70), AF was newly detected in 3.0% (*n* = 11), with increasing prevalence across AI risk categories. The non‐low‐risk group (moderate, high, and NA) had a significantly higher AF detection rate than the low‐risk group (OR 9.36, 95% CI 1.99–44.01). Subgroup analysis in those aged ≥65 years showed a similar trend (OR 8.09 [95%CI 1.63–39.7]). When the NA group (not eligible for AI) was excluded, similar trends were observed, although statistical significance was attenuated (OR 4.89 [95% CI 0.88–27.1] in the total, 5.09 [95% CI 0.89–29.0] in those aged ≥65 years). In the total cohort, AI‐ECG showed higher discriminative ability than the CHADS_2_ score ≥1 in both the total cohort (AUC 0.75 vs. 0.68) and participants aged ≥65 years (AUC 0.73 vs. 0.61).

**Conclusions:**

AI‐ECG risk determination correlated with AF detection in a Japanese healthy cohort, especially in the aged population, supporting its utility as a population‐based screening tool.

## Graphical abstract

AI‐ECG risk classification in a community‐based health screening program was associated with AF detection through 7‐day monitoring. Detection rates increased with risk level, especially in older adults, supporting its potential as a population‐based screening tool.

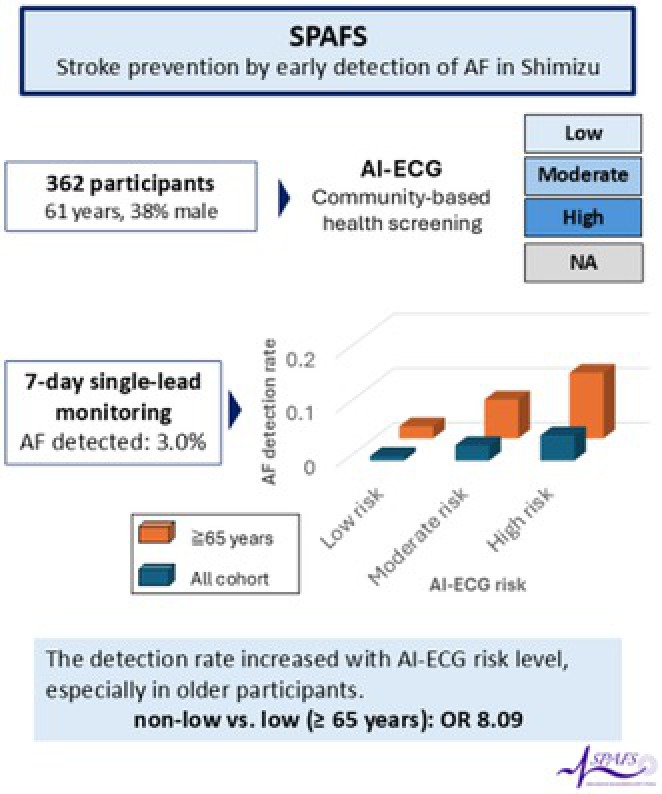

## 1 INTRODUCTION

Atrial fibrillation (AF) is the most common arrhythmia worldwide, and its prevalence is rising as the population ages.^1^ The overall AF detection rate is approximately 1%, which rises to 8%–10% in people aged >80 years.^2^, ^3^ However, only 2%–4% of cases can be detected through Japanese periodic health examinations.^4^, ^5^, ^6^ This underestimation occurs because patients with paroxysmal AF show no abnormalities on an electrocardiogram (ECG) when in sinus rhythm (SR). In the early stages of AF, symptoms are often minimal, making it challenging to detect during routine ECG screening.^7^ Because hidden AF may only be detected after a cardiogenic stroke,^8^ early detection and appropriate treatment may contribute to stroke prevention and prolonged healthy life expectancy. However, the current evidence on the net benefit of population‐based AF screening remains inconclusive. The U.S. Preventive Services Task Force (USPSTF) has stated that the evidence is insufficient to assess the balance of benefits and harms of routine AF screening.^9^ Nevertheless, it is important to recognize that, even if the overall effectiveness of screening remains unclear, certain individuals may derive substantial benefit from early identification of AF, as supported by evidence from targeted screening in high‐risk populations.^8^

Advances in artificial intelligence (AI) technology, developed using datasets of patients with cardiovascular disease, make it possible to detect hidden AF risk from an ECG taken during SR. An AI algorithm can identify subtle changes in the ECGs of patients with AF under SR.^10^, ^11^ However, it is unclear whether AI‐ECG, developed for patients with cardiovascular diseases, can prove valid in a population cohort with a low AF incidence. Although AI‐ECG has been reported as an effective screening tool for hidden AF in the United States,^12^, ^13^ no studies have assessed its efficacy in a healthy Japanese cohort.

In this article, we present that AI‐ECG, developed based on data from Japanese patients with cardiovascular diseases, can be used as a screening tool to reveal the risk of hidden AF in a healthy Japanese cohort in collaboration with local governments in Shizuoka prefecture. We conducted a prospective cohort study, “SPAFS” (stroke prevention by early detection of AF in Shimizu), a collaborative project of the Institute of Science Tokyo (formerly Tokyo Medical and Dental University), Shizuoka City, and the Shimizu Medical Association. Shimizu Ward, a part of Shizuoka City, which is located in the middle of Shizuoka prefecture, has a population of approximately 230,000, with an aging rate of 32.9% in 2020, which is slightly higher than the national average of 28.6%.^14^ Given its high aging rate, Shimizu Ward is an appropriate region for AF screening using AI‐ECG. Although the CHADS₂ score was originally developed to assess thromboembolic risk in patients with AF, previous studies have explored its utility in predicting AF.^15^ To further evaluate the potential utility of AI‐based ECG classification, we also compared its performance with that of the CHADS₂ score.

## 2 MATERIALS AND METHODS

### 2.1 Study design and participants

SPAFS is a prospective cohort study designed to assess the AI‐ECG risk distribution and AF detection rate through 7‐day ECG monitoring. The research protocol was approved by the Review Board at the Institute of Science Tokyo (M2021‐089).

Between January 2022 and July 2023, we enrolled 371 adult residents (≥40 years) of Shimizu Ward, Shizuoka City, who underwent medical health checkups at the Shimizu Medical Association Examination Center. Exclusion criteria were as follows: subjects who had already been diagnosed with AF, who underwent open chest surgery, and subjects who took antiarrhythmic drugs for any arrhythmias. Participation in the study was offered during the check‐in process, and written informed consent was obtained from those who agreed to participate. As only individuals who provided consent were included in the study, the total number of individuals who declined or were not approached was not systematically recorded.

At the Examination Center, the participants underwent a 12‐lead ECG with AI‐based AF risk classification. Additionally, routine annual health checkups were conducted to gather baseline variables (age, sex, body mass index (BMI), blood pressure, past medical history, smoking, and alcohol consumption). Standing height and body weight were measured in scrub suits without shoes. BMI was calculated as weight (kg) divided by height (m) squared. Blood pressure was measured twice in a sitting position after a few minutes of rest, and the average was recorded. Medical history, smoking, and alcohol consumption data were self‐reported by participants.

As the study was conducted as part of a population‐based screening, detailed symptom assessment was not performed. In general practice, individuals with strong or persistent symptoms are typically advised to seek medical attention rather than participate in routine checkups. Therefore, participants enrolled in this study were not expected to have experienced such symptoms at the time of screening. However, as eligibility was judged at the reception level, detailed information regarding prior symptoms was not systematically recorded.

The participants wore a single‐lead ECG for up to 7 days after registration. Those who exhibited AF on the baseline 12‐lead ECG were excluded from the 7‐day monitoring.

### 2.2 AI‐ECG model

We used prototype AI‐ECG machines developed by Fukuda Denshi (Tokyo, Japan), as reported previously.^16^ Briefly, AI‐ECG was developed as follows: To detect hidden AF signatures, a lightweight two‐dimensional (2D) convolutional neural network (CNN) model was utilized, consisting of four convolutional layers followed by dense layers with ReLU activation. Training and validation were conducted using data from cardiology outpatients, focusing on SR ECGs labeled for latent AF risk. The 12‐lead ECG input data during SR were preprocessed by R‐wave‐triggered averaging, transformed into a 2D binary image, and arranged into a composite layout with four rows and three columns to form the CNN input. Internal and external validations achieved areas under the curve (AUC) of 0.82 and 0.80, respectively, with sensitivities of 79.5% and 72.3%, and specificities of 77.8% and 77.7%. The model was implemented as standalone software and deployed on Fukuda Denshi ECG machines, enabling real‐time AF risk detection within approximately 2 seconds after ECG acquisition.

AI‐ECG assigns an AF risk score from 1 to 5, based on the estimated probability of AF. We categorized the risk as follows: “low risk” for AI classes 1 and 2, “moderate risk” for classes 3 and 4, and “high risk” for class 5. Participants whose baseline ECGs showed AF, atrial flutter (AFL), atrial tachycardia (AT), ectopic atrial rhythm, or any other rhythm deviating from SR were categorized as “NA: non‐applicable” (class 0), as the AI‐ECG model is designed for SR. For the primary analysis, we included the three risk‐classified groups (low, moderate, and high risk) to ensure valid comparisons.

We first described the overall distribution of AI‐ECG risk and participants' characteristics across the full screened population, including the NA group. Subsequently, for the primary analysis, we focused on participants with SR (i.e., those eligible for AI‐based risk classification) to compare baseline characteristics and AF incidence among the three risk groups (low, moderate, high). This approach allowed us to capture both the real‐world diversity of the screened cohort and the analytical performance of the AI risk classification.

### 2.3 Monitoring procedures

All participants wore a single‐lead Holter ECG (Duranta®, ZAIKEN, Tokyo, Japan)^17^ for up to 7 days. The monitoring data was transmitted to a cloud server via dedicated smartphones. To ensure monitoring quality, we set the inclusion criteria for monitoring time as at least 10 h per day for a minimum of 4 days. Participants who did not meet the monitoring criteria were excluded from the study.

### 2.4 Outcomes

The primary outcome was the newly diagnosed AF, defined as AF lasting 30 s or longer on single‐lead ECG, including cases identified on the baseline ECG. Monitoring data were automatically analyzed and double‐checked by the Institute of Science Tokyo laboratory staff, consisting of cardiologists and medical technologists. Only cases confirmed as AF were counted as outcome events, with cases identified as AFL or AT excluded. However, participants whose waveforms exhibited any arrhythmic abnormalities received individualized feedback recommending medical follow‐up, regardless of AF diagnosis.

We analyzed the AF detection rate across AI‐ECG risk categories. Subgroup analyses were also conducted for participants aged <65 and ≥65 years.

### 2.5 Patient follow‐up: “Shimizu model”

We established a regional system to ensure timely and appropriate treatment for participants with detected AF, facilitated through data sharing. The “Shimizu Model” includes data sharing among the Examination center, participants, primary care physicians, and cardiologists (Figure S1). Reports for each participant, including AI‐ECG risk classification, 7‐day monitoring results, and overall treatment recommendations, were sent from the examination center to all participants and primary care physicians. The system enables participants to visit a dedicated outpatient clinic in Shimizu Hospital's cardiovascular department, where they can receive care from cardiologists as needed. Treatment strategies were determined by the cardiologists according to the patient's CHADS_2_ score and overall clinical context. While we ensured that they were referred for appropriate management in accordance with guideline‐directed therapies by board‐certified cardiologists, the clinical outcomes of their treatment were not assessed as part of the primary endpoint.

Although this study was cross‐sectional in design and did not include longitudinal follow‐up, annual health checkups are routinely performed in this population, allowing for the potential assessment of changes in AF risk and detection in future analyses.

### 2.6 Statistical analysis

Statistical analyses were conducted using Stata software (version 16.1; StataCorp, College Station, Texas, USA). Statistical significance was based on a predetermined α of 0.05. Categorical variables were summarized as frequencies and percentages. Continuous variables were expressed as mean ± standard deviation (SD).

Baseline characteristics were compared across AI‐ECG risk groups using Kruskal–Wallis tests for continuous variables and chi‐squared tests for categorical variables. The CHADS_2_ score was calculated from the baseline data obtained at enrollment (congestive heart failure, hypertension, age ≥75 years, type 2 diabetes, and previous stroke/transient ischemic attack [doubled]).

Logistic regression was used to compare the AF detection rate between the low‐ and non‐low‐risk groups, with results expressed as odds ratios (OR) and 95% confidence intervals (CI). Subgroup analyses were performed by baseline age group (participants aged <65 years and those aged ≥65 years). There were no missing values in the dataset, and no data imputation was performed.

To evaluate the diagnostic performance of AI‐based risk classification and CHADS₂ score in predicting AF detection, we compared sensitivity, specificity, positive predictive value (PPV), and negative predictive value (NPV) for each binary classification. AI‐based risk was dichotomized as non‐low versus low, and the CHADS₂ score was categorized as ≥1 versus 0. These measures were calculated using the “diagt” command in Stata.

## 3 RESULTS

### 3.1 Baseline characteristics, AI‐ECG risk classification, and AF detection rate

A total of 371 residents participated in the study. No AF was found on the baseline 12‐lead ECG. Hence, all participants proceeded to 7‐day monitoring after the AI‐ECG. However, nine participants did not meet the monitoring time criteria. After applying the exclusion criteria, 362 participants were included in the final analysis.

The baseline characteristics for each risk category, including those not classifiable by the AI (NA group), are presented in Table 1. The mean age was 61.1 ± 10.5 years, with 38% (*n* = 137) males. The mean CHADS_2_ score was 0.49 ± 0.70. The prevalence rates of hypertension (*n* = 107, 30%), diabetes (*n* = 27, 7%), and stroke (*n* = 5, 1%) were also obtained. The AI‐ECG classified 66% (*n* = 239) as low‐risk, 10% (*n* = 36) as moderate‐risk, and 18% (*n* = 65) as high risk.

**TABLE 1:** Baseline characteristics and AI‐ECG risk category.

	Overall	High risk	Moderate risk	Low risk	NA	P value
	(*n* = 362)	(*n* = 65)	(*n* = 36)	(*n* = 239)	(*n* = 22)	
Age (years)	61.1 ± 10.5	61.5 ± 9.6	61.8 ± 8.8	60.5 ± 10.9	65.3 ± 10.3	0.3
Male (*n*)	137 (38%)	25 (38%)	20 (56%)	84 (35%)	8 (36%)	0.1
BMI (kg/m^2^)	22.6 ± 3.5	22.6 ± 3.5	23.4 ± 3.6	22.5 ± 3.6	21.9 ± 3.1	0.3
Systolic BP (mmHg)	123.9 ± 14.7	124.5 ± 16.3	122.2 ± 15.1	124.3 ± 14.0	121.4 ± 16.2	0.4
Diastolic BP (mmHg)	75.2 ± 10.7	76.1 ± 11.4	73.4 ± 10.2	75.6 ± 10.6	72.1 ± 11.1	0.4
Hypertension (*n*)	107 (30%)	18 (28%)	13 (36%)	64 (27%)	12 (55%)	0.04
Diabetes (*n*)	27 (7%)	4 (6%)	0 (0%)	20 (8%)	33 (14%)	0.2
Stroke (*n*)	5 (1%)	1 (1.5%)	1 (2.8%)	2 (0.8%)	1 (4.6%)	0.4
CAD (*n*)	12 (3.3%)	2 (3.1%)	3 (8.3%)	5 (2.1%)	2 (9.1%)	0.1
CHADS2 score	0.49 ± 0.70	0.45 ± 0.64	0.44 ± 0.65	0.46 ± 0.67	1.0 ± 1.07	0.08
Alcohol (≥20 g/day) (*n*)	85 (24%)	20 (31%)	12 (33%)	49 (21%)	4 (18%)	0.1
Smoking (*n*)	35 (9.7%)	4 (6.2%)	4 (11%)	22 (9.2%)	5 (23%)	0.1

*Note*: Data are mean ± SD, or *n* (%).

Abbreviations: BMI, body mass index; CAD, coronary artery disease; NA, not applicable.

Twenty‐two participants (6%) were classified as NA. The NA category included participants whose baseline ECGs were not in SR and therefore could not be analyzed using the AI‐based risk classification. Upon review of the ECG waveforms, the NA group comprised four cases with premature atrial contractions, eight with premature ventricular contractions, five with ectopic atrial rhythm, and five cases with non‐SRs that could not be classified due to poor signal quality or ambiguous findings.

The medians of the baseline variables were similar across the risk categories, except for the prevalence of hypertension, which was higher in the NA than in the other three risk categories. However, when limiting the analysis to the three AI‐classified groups, no significant difference in the prevalence of hypertension was found (*p* = 0.5).

**FIGURE 1:**
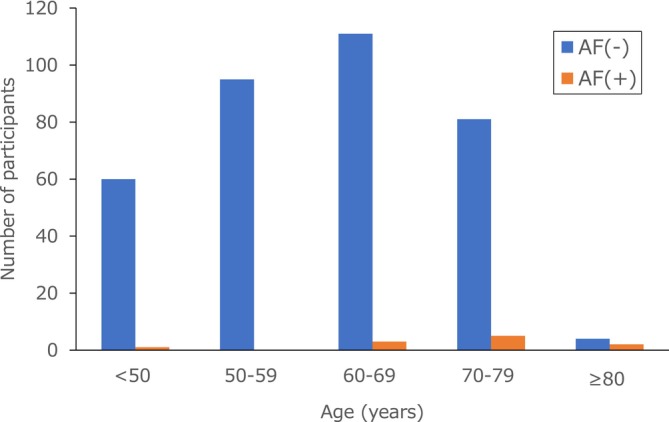
Age‐specific distribution of AF detections. Blue bars indicate the number of undetected cases, and orange bars represent the number of detected cases within each age group. The 60–69 age group had the largest number of participants across the age categories. The higher AF detection rate was noted in older age groups.

Figure 1 shows the age distribution of all the participants and the AF detection rate of the single‐lead ECG divided by 10 years of age. The 60–69 age group had the largest number of participants, followed by the 50–59 and 70–79 age groups. The overall AF detection rate was 3.0% (*n* = 11). The detection rate of AF increased with age (60–69 years old: 2.6%, 70–79 years old: 5.8%; and >80 years, 33.3%).

**FIGURE 2:**
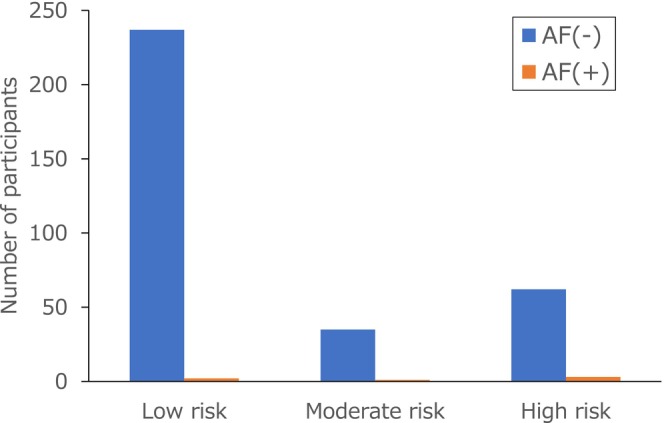
Distribution of AF detections across AI‐ECG risk classifications. Blue bars represent cases without AF, and orange bars represent those with newly detected AF, stratified by AI‐ECG risk classification (low, moderate, high). Participants categorized as NA were excluded from this analysis (NA, not applicable).

Figure 2 shows the AF detection rates according to AI‐ECG risk categories. Based on 7‐day single‐lead ECG monitoring, AF was detected in 2 of 239 participants with low risk (0.8%), 1 of 36 with moderate risk (2.8%), and 3 of 65 with high risk (4.6%). In the NA group, 5 of 22 patients had AF (22.7%, Table S1).

When the non‐low‐risk is defined as the aggregate of the three groups other than the low‐risk group—that is, moderate‐risk, high risk, and NA—the prevalence of AF in the non‐low‐risk group was significantly higher than the low‐risk group (low vs. non‐low: 0.8% vs. 7.3%, odds ratio 9.36 [95%CI 1.99–44.01], *p* = 0.0009). When participants in the NA group were excluded, the odds of AF detection remained higher in the non‐low‐risk group than in the low‐risk group (0.8% vs. 4.0%), although the difference did not reach statistical significance (odds ratio 4.89 [95%CI 0.88–27.1], *p* = 0.07).

We compared the diagnostic performance of AI‐based risk classification (non‐low vs. low) and CHADS₂ score in predicting AF detection. The AI classification demonstrated a sensitivity of 81.8%, specificity of 67.5%, PPV of 7.3%, and NPV of 99.2%, with an area under the ROC curve (AUC) of 0.75. When limiting the analysis to participants with SR, who were eligible for AI‐based risk stratification, the sensitivity decreased to 66.7%, specificity increased to 71.0%, PPV was 4.0%, NPV was 99.2%, with an AUC of 0.69.

The CHADS₂ score (≥1 vs. 0) showed slightly lower performance in both analyses. In the full cohort, the CHADS_2_ score yielded a sensitivity of 72.7%, specificity of 62.4%, and PPV of 5.7%, NPV of 98.6%, and an AUC of 0.68. In the SR‐only analysis, its sensitivity was 66.7%, specificity 63.2%, PPV 3.1%, NPV 99.1%, and AUC 0.65.

### 3.2 Subgroup analysis by age

To assess the combined impact of age and AI‐ECG categories, participants were divided into two age groups: those aged <65 years (*n* = 219) and those aged ≥65 years (*n* = 143). Among those aged <65 years, only one AF was detected, which occurred in the NA group (0.46%). Given that only one AF case was identified among participants aged <65 years, further analysis was not performed in this subgroup due to the limited statistical interpretability.

**FIGURE 3:**
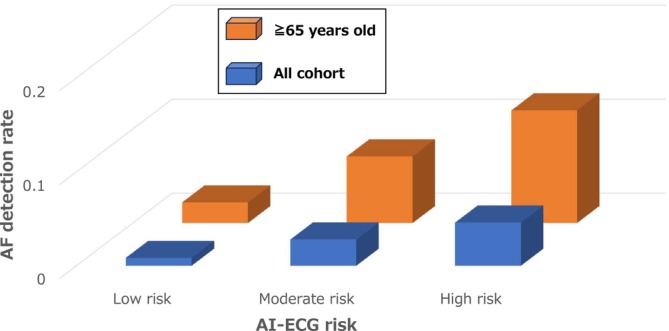
Detection rates of AF (proportion) within each risk classification (low, moderate, and high) for all cohorts (blue bars) and participants aged 65 years and older (orange bars). The detection rate of AF increased with rising risk levels. This pattern was particularly evident among participants aged 65 years and older. Participants categorized as NA were excluded from this analysis (NA, not applicable).

As the remaining 10 AF patients were included in the group aged 65 years and older, the AF detection rate for those aged ≥65 years was raised to 7.0%. The detection rates were 2.2%, 7.1%, and 12.0% in the low‐, moderate‐, and high‐risk groups (Figure 3). In the NA group, the AF detection rate was 30.8% (Table S1). The non‐low‐risk group had a significantly higher prevalence of AF than the low‐risk group (low vs. non‐low: 2.2% vs. 7.1%, OR 8.09 [95%CI 1.65–39.7], *p* = 0.003) (Table 2). The association did not reach statistical significance when the NA group was excluded (OR 5.09, 95%CI 0.89–29.0, *p* = 0.067), but the trend remained.

**TABLE 2:** Distribution of AF detections by AI‐based risk classifications in participants ≥65 years.

AI risk	Participants with AF (*n*)	Participants without AF(*n*)		Odds ratio [95% CI]	P value
Low	2	89		1	
Moderate	1	13	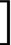	8.09 [1.65–39.7]	0.003
High	3	22			
NA	4	9			

In this subgroup, both the AI‐based risk classification and the CHADS2 score (≥1 vs. 0) showed comparable sensitivity (80.0%). However, the AI‐based model demonstrated higher specificity than the CHADS_2_ score (66.9% vs. 41.4%), PPV (15.4% vs. 9.3%), NPV (97.8% vs. 96.5%), and overall discrimination (AUC = 0.73 vs. 0.61), indicating better screening performance in older adults.

When the NA group was excluded, similar trends were observed as in the total cohort: sensitivity decreased, while specificity increased, and the AI model maintained superior discriminative ability compared with CHADS_2_ (AUC = 0.69 vs. 0.55). The NPV remained high (97.8%) in this analysis.

### 3.3 Patient follow‐up

We followed up the patients using the “Shimizu Model,” as previously described. All participants with AF visited the SPAFS outpatient clinic at Shizuoka City Shimizu Hospital or cardiology clinics, both staffed by board‐certified cardiologists. Referral completion was 100%, ensuring that all diagnosed individuals were linked to specialist care. Although they were appropriately treated according to the Japanese Heart Rhythm Society guidelines, specific treatment details were not collected or analyzed in this study.

## 4 DISCUSSION

This is the first study in Japan to apply AI‐based ECG analysis on a population‐wide scale as part of routine health checkups. This novel implementation allows for the evaluation of AI‐ECG screening performance in a real‐world, asymptomatic cohort with broad age and risk distributions. This prospective study demonstrated that AI‐ECG can evaluate hidden AF risk in annual health checkup settings in a healthy Japanese cohort with a low prevalence of AF. The mean age of the participants in our study was 61 years, and they were monitored for a duration of 7 days. Under these conditions, AF was detected in 0.8% of low‐risk patients and 7.3% of non‐low‐risk patients (including the NA group). The non‐low‐risk group had a 9.36‐fold higher AF detection rate than the low‐risk group. In contrast, a previous study investigating individuals with stroke risk factors but without a diagnosis of AF identified an AF detection rate of 1.6% in low‐risk patients using AI‐based ECG analysis and 7.6% in high‐risk patients following an average monitoring duration of 22.3 days.^13^ In contrast to the previous study, our study targeted a healthy population and limited the monitoring duration to 7 days. Despite differences in background populations and the duration of monitoring, the detection rates observed in the present study were comparable to those reported previously, reinforcing the utility of AI‐ECG even in the primary prevention cohort. Also, it should be noted that the AI‐ECG model was generated using ECG data mostly in subjects who regularly visited hospitals, but not in the healthy population.^16^ This study showed that the AI‐ECG model showed some precision in a healthy population.

The comparison of AI‐based risk classification and CHADS₂ score revealed that the AI model had superior diagnostic performance in terms of both sensitivity and overall discrimination (AUC = 0.75 vs. 0.68). While the PPV of both models was limited due to the low prevalence of AF in the population, the high NPV supports the potential utility of AI‐based classification as a first‐line screening tool to rule out low‐risk individuals in community settings. However, when the analysis was limited to participants with SR, the group eligible for AI‐based risk scoring, the diagnostic performance of the AI model was slightly reduced (AUC 0.69). These findings should be interpreted with caution, particularly given the low prevalence of AF in the overall cohort, especially among younger participants.

The subgroup analysis revealed that for those aged ≥65 years, AI‐ECG risk determination effectively reflected the AF detection rate. The analysis demonstrated that the AI‐based classification maintained high sensitivity while achieving superior specificity and diagnostic accuracy compared with the CHADS2 score, supporting its potential value as a screening tool in older populations. However, when the NA group was excluded, the AUC of the AI model decreased from 0.73 to 0.69, and the OR for AF detection between low‐ and non‐low‐risk categories did not reach statistical significance. Nevertheless, the overall trend of increased AF detection with higher risk categories remained consistent, and the model retained a high NPV (97.8%), suggesting continued utility as a rule‐out tool in this age group. In contrast, for patients aged <65 years, AF was found only in the NA group, not in any of the other categories. Hence, in our cohort, it was impossible to present a difference in the AF detection rate by AI‐ECG risk determination in patients aged <65 years with SR ECG. However, the observed risk differences in the younger group may potentially serve as indicators of the future risk of AF. Longitudinal follow‐up of the same cohort may produce differences in the AF detection rate. These findings highlight the complementary strengths and limitations of AI‐ECG and CHADS2 score‐based screening approaches, depending on the target population and age distribution.

Among individuals aged ≥65 years, AF was detected even in those classified as low risk. While the detection rate in this group remains very low (0.8% of the overall population and 2.2% within the subset aged ≥65 years), the possibility of AF cannot be entirely excluded, even in patients deemed low risk. However, from the perspective of screening within health examinations, it is essential to ensure cost‐effectiveness by concentrating on a more precisely defined population, specifically those with a higher risk profile from a public health perspective. Combining the population‐based approach with AI‐ECG and the high‐risk approach with 7‐day monitoring can make AF detection more sophisticated than the current AF screening in Japanese periodic health examination settings. It is expected that the integration of currently available clinical scores^18^, ^19^, ^20^ for AF prediction with AI‐based models, as well as the development of additional modalities, will contribute to enhanced predictive capabilities.

The management of the NA group remains a topic of debate, as this population is characterized by the presence of ECG abnormalities detectable through visual inspection without AI, and thus falls outside the scope of AI‐based classification. Consequently, the NA group could reasonably be excluded when evaluating the intrinsic performance of the AI algorithm, as it was primarily trained on sinus ECGs. Nevertheless, considering that this was the first AI‐ECG project conducted within the context of Japan's public health checkup system, it was necessary to evaluate the AF detection rate across the entire cohort, including those not classifiable by the model. These findings suggest that adopting a targeted approach for all individuals identified as having a risk level other than “low” during AI‐ECG‐based medical checkups may represent an effective strategy for enhancing the efficiency of AF detection in the screenings. In this context, AI‐ECG can still serve as a valuable first‐step screening tool in the context of population‐based health checkups.

Our study had several limitations. First, the first‐year participants may not reflect the entire risk distribution in the region. The detection rate of AF in participants aged <65 years was very low, making it challenging to interpret the risk assessment results. The proportion of older adults (≥65 years) in our cohort was relatively low, which may have limited the overall detection rate of AF. Given that AF prevalence increases substantially with age, the inclusion of a larger number of elderly participants may enhance the screening yield in future studies.

Second, the duration of monitoring remains controversial. Although we selected 7 days as the monitoring duration within a practically achievable period in this study, further research is needed to optimize the monitoring time. The AF detection rate and monitoring time exhibit a trade‐off relationship. A more extensive evaluation can lead to higher AF detection rates.^8^, ^21^ Theoretically, wearable devices designed for continuous and lifelong monitoring would provide the most reliable method for detecting AF. However, the practical implementation of such devices currently depends on individual management and requires the active cooperation of the user. Although longitudinal follow‐up was not conducted in this study, annual health checkups are routinely performed in the study population, which may enable future assessment of year‐to‐year changes in AF risk and detection.

Moreover, challenges related to cost, data management, and the responsibility for integrating such devices into health checkup programs administered by local governments pose significant barriers. Third, the appropriateness of the treatment indications remains uncertain, especially for patients detected at an extremely early stage through AI technology. We established a regional network, the Shimizu Model, to bridge the gaps between AF screening, detection, and treatment. As a result, in this study, all participants with newly detected AF successfully visited cardiology clinics, achieving a 100% referral rate. Although specific treatment modalities were not within the scope of this study and were not collected, all diagnosed individuals were linked to specialist care in accordance with the Japanese Heart Rhythm Society guidelines. However, these current guidelines were not developed with individuals identified through AI‐based screening. Therefore, it remains unclear whether the treatment indications for early stage AF should align with the current standards.^22^ Although the indications for invasive procedures, including catheter ablation, are controversial, anticoagulation therapy based on the CHADS2 score should be reasonable, regardless of the AF stage, to prevent stroke and contribute to extending healthy life expectancy.

## 5 CONCLUSION

We demonstrated that the AF risk assessed by AI‐ECG correlates with the AF detection rate from 7‐day monitoring in a Japanese prospective cohort with healthy subjects. Further studies are needed to confirm its efficacy and enhance its validity as a screening tool.

## FUNDING INFORMATION

This study was supported by Shizuoka City, Shimizu Medical Association, Japan Agency for Medical Research and Development (no. JP19he2102002, to T.F.), and Japan Science and Technology Agency (no. JPMJPF2101).

## CONFLICT OF INTEREST STATEMENT

Dr. Yoshinaga declares a conflict of interest as the Representative Director of everMedica Co., Ltd., a for‐profit organization. All other authors declare no conflicts of interest for this article.

## ETHICS STATEMENT

The research protocol was approved by the Review Board at the Institute of Science Tokyo (formerly Tokyo Medical and Dental University, M2021‐089).

## PATIENT CONSENT STATEMENT

All participants gave written informed consent to the study.

## Supporting information


**Table S1.** Prevalence of AF by risk category in all participants and aged ≥65 years.

**Figure S1.** Diagram of the regional network in Shimizu ward after the SPAFS visit, showing connections between participants, primary care facilities, specialized healthcare providers, as well as referral pathways.

## Data Availability

The identified participant data will not be shared.
