# Supplementary figures and images for: AI‐ECG for early detection of atrial fibrillation: First‐year results from a stroke prevention study in Shimizu, Japan
Source: J Arrhythm. 2025 Jul 4;41(4):e70132. doi: 10.1002/joa3.70132 (PMC12227326; doi:10.1002/joa3.70132)

**Supplementary Figure.**

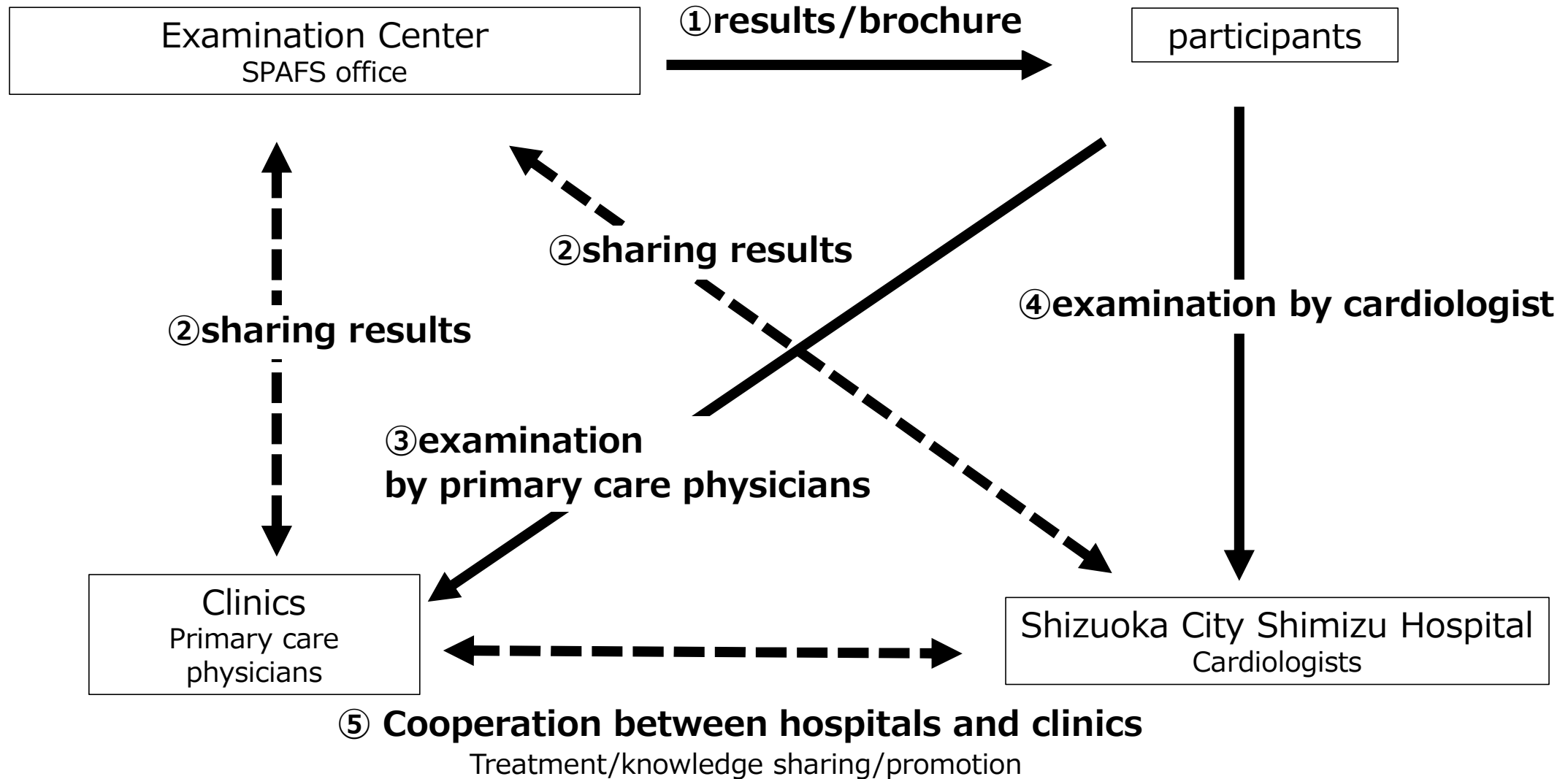

Supplement: Supplementary file 2 — Figure S1. Diagram of the regional network in Shimizu ward after the SPAFS visit, showing connections between participants, primary care facilities, specialized healthcare providers, as well as referral pathways. [file JOA3-41-e70132-s001.pdf]
